# Activation of cell-free mtDNA-TLR9 signaling mediates chronic stress-induced social behavior deficits

**DOI:** 10.1038/s41380-023-02189-7

**Published:** 2023-08-01

**Authors:** Ashutosh Tripathi, Alona Bartosh, Carl Whitehead, Anilkumar Pillai

**Affiliations:** 1 Pathophysiology of Neuropsychiatric Disorders Program, Faillace Department of Psychiatry and Behavioral Sciences, The University of Texas Health Science Center at Houston (UTHealth), Houston, TX USA; 2 Department of Psychiatry and Health Behavior, Augusta University, Augusta, GA USA; 3 Charlie Norwood VA Medical Center, Augusta, GA USA

**Keywords:** Neuroscience, Physiology

## Abstract

Inflammation and social behavior deficits are associated with a number of neuropsychiatric disorders. Chronic stress, a major risk factor for depression and other mental health conditions is known to increase inflammatory responses and social behavior impairments. Disturbances in mitochondria function have been found in chronic stress conditions, however the mechanisms that link mitochondrial dysfunction to stress-induced social behavior deficits are not well understood. In this study, we found that chronic restraint stress (RS) induces significant increases in serum cell-free mitochondrial DNA (cf-mtDNA) levels in mice, and systemic Deoxyribonuclease I (DNase I) treatment attenuated RS-induced social behavioral deficits. Our findings revealed potential roles of mitophagy and Mitochondrial antiviral-signaling protein (MAVS) in mediating chronic stress-induced changes in cf-mtDNA levels and social behavior. Furthermore, we showed that inhibition of Toll-like receptor 9 (TLR9) attenuates mtDNA-induced social behavior deficits. Together, these findings show that cf-mtDNA-TLR9 signaling is critical in mediating stress-induced social behavior deficits.

## Introduction

Stressful experiences are part of day-to-day life, but chronic stress conditions have negative impact on our health and well-being. Chronic stress has been implicated in the pathophysiology of many neuropsychiatric disorders, especially in anxiety, mood disorders and post-traumatic stress disorder (PTSD) [1, 2]. Significant impairments in social behaviors and reductions in social cognition and social motivation have been reported in these disorders [3, 4]. In addition, rodent studies have shown impairments in social behavior such as reductions in social interaction following chronic stress conditions [5–11]. Furthermore, chronic stress-induced changes in social behavior are associated with reductions in molecules that play important roles in excitation/inhibition balance, and synaptic function [12–15]. However, the mechanism that links chronic stress to social behavior deficits is not well understood. It is known that systemic administration of antigens results in range of behavioral and mood changes in humans [16–20]. In addition, chronic stress conditions are associated with increased inflammatory responses in both humans and animal models [20–28]. In rodents, chronic unpredictable stress [20, 21], chronic adolescent stress [22], and repeated social defeat [23] have been shown to induce increases in proinflammatory markers in the central nervous system (CNS) and periphery. Also, repeated social defeat resulted in the infiltration of peripheral monocytes into the brain which were associated with anxiety-like behavior in mice [26, 29, 30].

Stressful conditions are also known to alter mitochondrial components, mitochondrial energy production capacity and mitochondrial morphology [31–33]. The mitochondrial DNA (mtDNA) is deficient in histones and effective repair mechanisms and therefore, mtDNA may be more prone to stress-induced damages [34–37]. Accordingly, stress conditions promote mtDNA release from the mitochondria to the cytosol or extracellular space [35]. The circulating cell-free mtDNA (cf-mtDNA) is known to activate Toll-like receptor 9 (TLR9) signaling which results in the activation of several proinflammatory cytokines [38]. Chronic stress has been shown to increase blood mtDNA levels in mice [37, 39]. In clinical studies, increased levels of blood mtDNA have been found in individuals who had endured either parental loss or childhood maltreatment and with psychopathology including major depression [40]. Higher blood mtDNA have also been found in subjects with clinical levels of depressive symptoms [41–44] and in suicide attempters [45]. Since mtDNA is associated with inflammation [46–48], it is important to understand the role of mtDNA in chronic stress-induced inflammation and behavioral effects.

Under physiological conditions, mitochondrial removal and replenishment are well balanced to maintain the mitochondrial function [49]. Mitophagy, selective autophagy of mitochondria, is a cellular mechanism that eliminates damaged mitochondria. It is known that impaired mitophagy process leads to the release of mtDNA into the cytoplasm and out into the extracellular space resulting in an elevated release of proinflammatory cytokines [50–53]. Among the various mitophagy-related molecules that protect mitochondria from pathogens and damage, mitochondrial antiviral-signaling protein (MAVS) is an integral part of the cellular stress response [54]. While MAVS plays a key role in maintaining mitochondrial homeostasis via autophagy [55] and is involved in propagating antiviral responses in the brain [56, 57], chronic activation of this pathway can result in inflammation-driven pathology [58–61].

In the present study, we investigated the role of mtDNA in mediating chronic stress-induced increases in neuroinflammation and social behavioral deficits in mice. Also, we examined whether inhibition of MAVS and TLR9 pathways could attenuate stress-induced changes in social behavior.

## Materials and methods

### Animals

Adult male CD1 mice, male C57BL/6 J mice, male TLR9^-/-^ mice (TLR9 KO; strain #034449), male and female MAVS^-/-^ mice (MAVS KO; strain #008634) of 8 weeks old and their age-matched wild type (WT) controls were purchased from The Jackson Laboratory. Animals were housed in the animal facility at The University of Texas Health Science Center at Houston or Augusta University. Mice were housed and maintained (5 mice per cage) in standard polypropylene cages in a 12-h light-dark cycle in compliance with the US National Institute of Health guidelines, which was approved by The University of Texas Health Science Center at Houston and Augusta University animal welfare guidelines. Mice were assigned to experimental groups based on their genotype. Mice were selected randomly in a blinded manner to perform the experiments.

### Restraint stress (RS) procedure

In the RS paradigm, mice were restrained in well-ventilated 50 ml Falcon tubes for 2 h/day for 21 consecutive days. Control mice were housed under normal conditions in the usual cages. Mice were tested for behavior the day following the last restraint session.

### Deoxyribonuclease I (DNase I) and rapamycin (RAPA) treatment

Mice were intraperitoneally (i.p.) injected with DNase I (100U; Sigma–Aldrich, MO, USA), RAPA (10 mg/kg; Sigma–Aldrich) or vehicle (PBS) twice a week during the RS paradigm.

### Mitochondrial DNA (mtDNA) purification and treatment

Mitochondria were isolated from the spleen of mice exposed to RS using a mitochondria isolation kit (#89801; Thermo Fisher Scientific, MA, USA) following the manufacturer’s instructions. mtDNA were purified from mitochondrial pellets using DNeasy blood and tissue kits (Qiagen, Hilden, Germany). The DNA concentration and purity were examined by spectrophotometry. The endotoxin levels in the DNA samples were determined using the Limulus amoebocyte lysate assay (Thermo Scientific). For mtDNA treatment, WT and TLR9 KO mice were injected once with mtDNA (30 µg/mouse; i.p.) or vehicle (PBS) and behavior tests were performed at 4 h later. The above mtDNA concentration has been shown to induce systemic inflammation [62].

### Cell-free mitochondrial DNA (cf-mtDNA) analysis by qRT-PCR

Total DNA was isolated from 250 μl serum using DNA isolation/purification kits (DNeasy blood and Tissue kit; Qiagen, Hilden, Germany) according to the manufacturer’s protocol. Quantity of mtDNA was measured by qRT-PCR by analyzing mitochondrially encoded 12 S ribosomal RNA and cytochrome c oxidase I (Cox1) genes. Primers were synthesized by Integrated DNA Technologies. MasterCycler (Eppendorf, Westburg, NY, USA) was used to perform qRT-PCR using iTaqTM Universal SYBR® Green Supermix (Bio-Rad, CA, USA). Mitochondrial DNA levels were adjusted for nuclear DNA levels using 18 S rRNA expression and analyzed using the ΔCT method [62, 63].

### Behavioral tests

Mice were tested for behavior in a room with ambient temperature, lighting, pressure and sound. Mice were habituated to the behavior rooms in their home cages 1 h prior to the testing. All behavioral tests were performed and scored blind to the treatment.

### Three-chamber test

The three-chamber test was performed using an apparatus made of clear Plexiglas® with dimension of 19 cm × 45 cm × 22 cm. Two openings provided access to each compartment. Two identical wire containers were placed in the two side chambers. Test mouse was placed vertically in the apparatus’s middle chamber and allowed to move freely for initial 5 min to get habituated. Age, sex, and background-matched stranger mouse was then introduced in the one of the wire containers. The ventilated wire container allowed air exchange, but prevented direct physical contact. The time spent by test mouse in the different chambers (stranger mouse chamber, middle chamber and empty chamber) was video recorded for another 5 min.

### Reciprocal social interaction test

In this test, the test mouse was allowed to move freely across the entire 3-chamber apparatus to interact physically with the age, sex, and background-matched stranger mouse for 5 min. The interaction between the mice was defined as close physical contact, nose-to-nose sniffing, ano-genital sniffing, and grooming. Time of interaction (initiated by the test mouse only) was video recorded for 5 min and scored blindly.

### Flow cytometry

For the flow cytometry analysis, mouse whole blood was collected in heparin tube (#366667; BD, NJ, USA) after decapitation under anesthesia. RBC lysis buffer was added to lyse RBC from the whole blood according to manufacturer’s protocol (#420301; BioLegend, CA, USA). Following washing with PBS, cells were incubated with flow antibodies, CD11b (clone M1/70, 1:300), Ly6C (clone HK1.4, 1:300) from BioLegend, CA, USA and TLR9 (clone 26C593.2, 1/300) from Novus Biologicals, LLC, CO, USA. Cells were washed with PBS and fixed with fixation buffer (Affymetrix eBioscience). Samples were run in BD FACSAriaII instrument and were analyzed using BD FACSDiva software (BD Biosciences, San Jose, CA). Fluorescence Minus One (FMO) controls were used to accurately identify the positive cell populations for each marker. Specific markers on the cells were reported as the percentage of the number of gated events. Median fluorescence intensity derived from a fluorescence graph was used to study the level of cell surface expression.

### Quantitative reverse transcriptase PCR (qRT-PCR)

For the qRT-PCR analyses, the prefrontal cortex (PFC) tissues from mice were collected immediately following decapitation under anesthesia, according to a mouse brain atlas [21]. Total RNA from the PFC samples was isolated by using a commercially available kit (SV RNA Isolation, Promega, Madison, WI, USA). MasterCycler (Eppendorf, Westburg, NY, USA) was used to perform qRT-PCR using iTaq^TM^ Universal SYBR® Green Supermix (Bio-Rad, CA, USA). Primers specific to genes were synthesized by Integrated DNA Technologies. Housekeeping gene (beta2-microglobulin (*B2m*)) was used to normalize the gene of interest. A list of primers used is given in Table S1.

### Statistical analysis

No statistical methods were used to predetermine sample size, but our sample sizes were similar to those reported in previous study [28]. Data were presented as mean ± SEM. Statistical analysis were done using two-tailed Student’s *t*-tests to compare two-group or Analysis of Variance (ANOVA) for the multiple-group comparisons. Grubbs’ test was performed to identify the significant outlier by using GraphPad online calculator [https://www.graphpad.com/quickcalcs/Grubbs1.cfm]. Bonferroni’s post hoc test was performed using GraphPad Prism 9.0.0 and *p* < 0.05 was considered significant.

## Results

### DNase I treatment attenuates chronic stress-induced social behavior deficits and increased inflammatory markers in the PFC

**Fig. 1:**
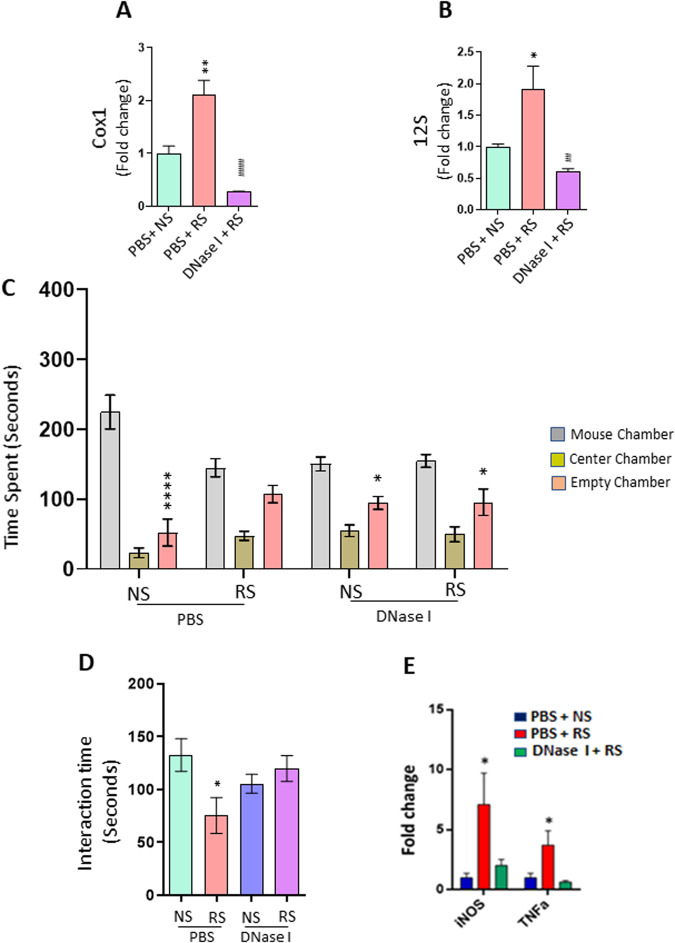
DNase I treatment attenuates chronic stress-induced social behavior deficits, and increased inflammatory markers in the PFC. **A**, **B** Expression of serum mtDNA genes (Cox1 and 12 S) in no stress (NS) or restraint stress (RS) exposed mice treated with PBS or DNase I. Cox1; One-way ANOVA, **p < 0.01 (vs PBS + NS) and ####*p* < 0.0001 (vs PBS + RS), *n* = 4 per group. 12 S; One-way ANOVA, *p < 0.05 (vs PBS + NS) and ##*p* < 0.01 (vs PBS + RS), *n* = 4 per group. **C**, **D** DNase I treatment attenuated RS-induced deficits in social behavior. **C** Time in chamber in the three-chamber social interaction test. Two-way ANOVA, chamber (F (2, 81) = 87.81; *p* < 0.0001); interaction (chamber X treatment) (F (6, 81) = 6.174; *p* < 0.0001). *p < 0.05 and ****p < 0.0001 (mouse chamber vs empty chamber); *n* = 7–8 per group. **D** Reciprocal social interaction test; Two-way ANOVA, interaction (stress x treatment) (F (1, 23) = 6.575, *p* = 0.0173); *p < 0.05 vs PBS + NS; *n* = 6–7 per group. **E** mRNA expressions of proinflammatory cytokines in the PFC of RS mice treated with PBS or DNase I. iNOS; One-way ANOVA, *p < 0.05 (vs PBS + NS) *n* = 4–5 per group. TNFα; One-way ANOVA, *p < 0.05 (vs PBS + NS) *n* = 4–5 per group.

To determine the role of cf-mtDNA in mediating RS-induced social behavior deficits, we first measured the levels of cf-mtDNA in the serum of mice exposed to RS. The levels of two mtDNA genes, cytochrome c oxidase subunit I (Cox1) and mitochondrial 12 S rRNA gene (12 S) were examined. qPCR results showed significant increases in the expression of Cox1 and 12 S in the serum of mice exposed to RS (Fig. 1A, B). To examine the role of cf-mtDNA on RS-induced changes in social behavior, we performed three-chamber sociability and reciprocal social interaction tests in mice treated with DNase I during NS or RS exposure. DNase I is known to deplete cf-mtDNA [64, 65]. NS mice spent more time in the stranger mouse chamber than the empty cage chamber, whereas RS mice showed no preference for either chamber (Fig. 1C). However, RS mice injected with DNase I spent more time in the chamber housing stranger mouse than the empty cage chamber (Fig. 1C). In the reciprocal social interaction test, RS mice showed decreased interaction with a stranger mouse when compared with NS mice (Fig. 1D). Interestingly, treatment with DNase I significantly attenuated RS-induced decrease in interaction time in mice (Fig. 1D). Furthermore, we found that DNase I treatment significantly attenuated RS-induced increases in proinflammatory markers, iNOS and TNFα in the PFC, a key brain region implicated in social behavior (Fig. 1E). These results suggest that cf-mtDNA plays a critical role in RS-induced social behavior deficits and neuroinflammation.

### Rapamycin treatment attenuates chronic stress-induced increase in serum cf-mtDNA and social behavior deficits

**Fig. 2:**
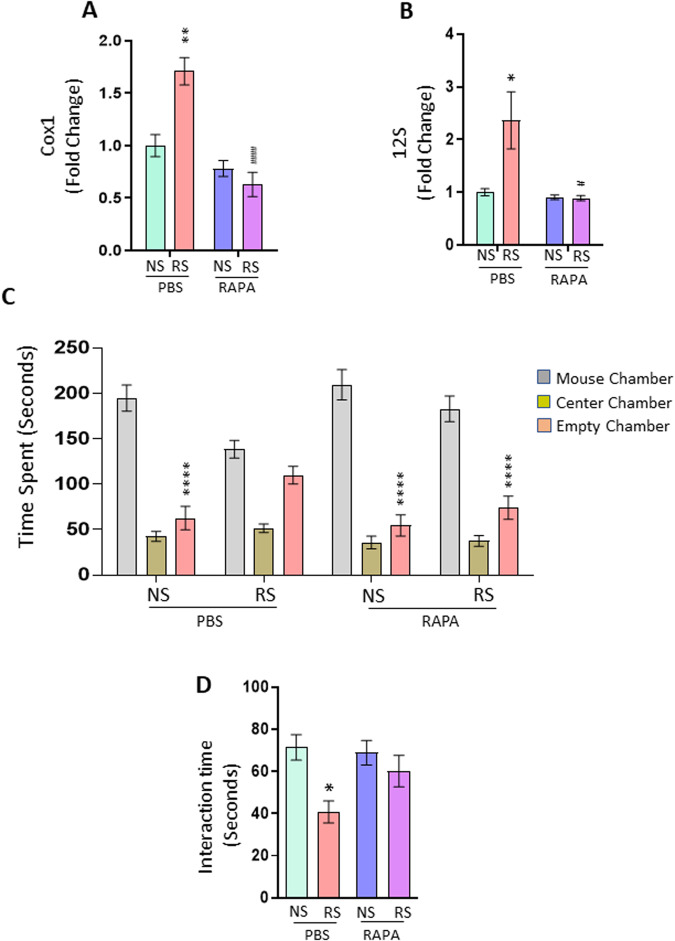
Rapamycin treatment attenuates chronic stress-induced increase in serum cf-mtDNA and behavior deficits. **A**, **B** Expression of serum mtDNA genes (Cox1 and 12 S) in no stress (NS) or restraint stress (RS) exposed mice treated with PBS or Rapamycin (RAPA). Cox1; Two-way ANOVA, stress (F (1, 12) = 6.493, *p* = 0.0256); treatment (F (1, 12) = 35.32, *p* < 0.0001) and interaction (stress x treatment) (F (1, 12) = 15.55, *P* = 0.0019); **p < 0.01 (vs PBS + NS) and ####*p* < 0.0001 (vs PBS + RS), *n* = 4 per group. 12 S; Two-way ANOVA, stress (F (1, 12) = 5.953, *p* = 0.0312); treatment (F (1, 12) = 8.141, *p* = 0.0145) and interaction (stress x treatment) (F (1, 12) = 6.258, *p* = 0.0278); *p < 0.05 (vs PBS + NS) and #*p* < 0.05 (vs PBS + RS), *n* = 4 per group. **C**, **D** RAPA treatment attenuated RS-induced deficits in social behavior. **C** Time in chamber in the three-chamber social interaction test. Two-way ANOVA, chamber (F (2, 93) = 172.3, *p* < 0.0001); interaction (chamber X treatment) (F (6, 93) = 6.635, *p* < 0.0001). ****p < 0.0001 (mouse chamber vs empty chamber); *n* = 8–9 per group. **D** Reciprocal social interaction test; Two-way ANOVA, treatment (F (1, 31) = 8.976, *p* = 0.0053); *p < 0.05 vs PBS + NS; *n* = 8–9 per group.

It is known that impaired mitophagy leads to the accumulation of dysfunctional mitochondria and mtDNA leakage to the cytosol whereas activation of mitophagy reduces inflammation by clearing damaged mitochondria [66]. Rapamycin (RAPA) is an activator of autophagy/mitophagy [67, 68]. Using the PBS-treated mice as control group, we examined whether RAPA treatment could attenuate RS-induced social behavior deficits and increases in cf-mtDNA levels. Gene expression data show that RAPA treatment significantly attenuated RS-induced increases in Cox1 and 12 S levels (Fig. 2A, B). To examine the effects of RAPA on chronic stress-induced changes in social behavior, we performed three-chamber sociability and reciprocal social interaction tests in mice treated with RAPA. RS mice injected with RAPA spent more time in the chamber housing stranger mouse than the empty cage chamber (Fig. 2C). Furthermore, RAPA treatment significantly attenuated RS-induced decrease in interaction time in mice (Fig. 2D).

### Chronic stress-induced changes in cf-mtDNA levels and social behavior deficits are MAVS-dependent

**Fig. 3:**
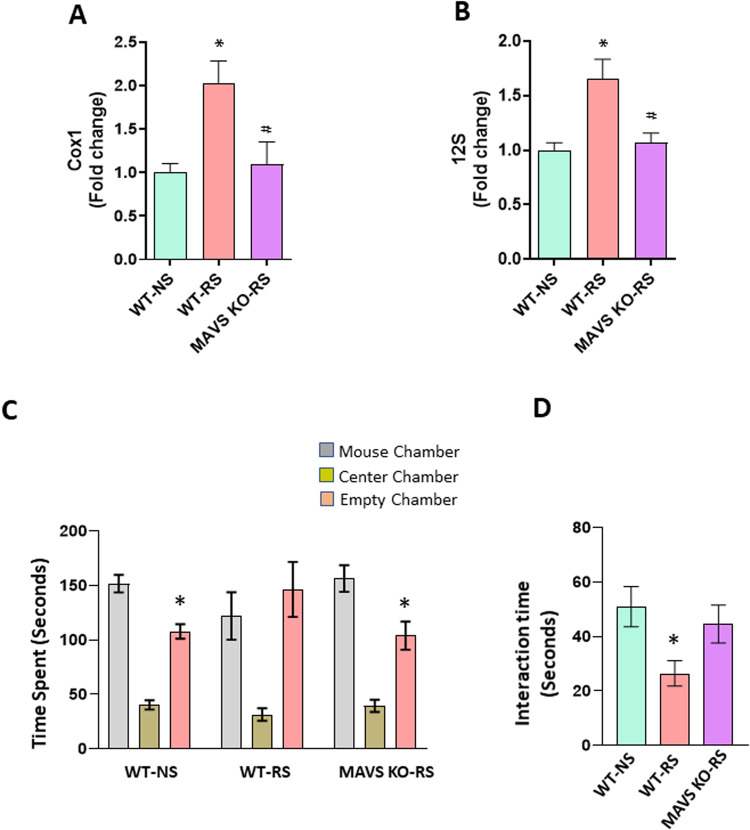
Chronic stress-induced changes in serum cf-mtDNA and social behavior deficits are MAVS-dependent. **A**, **B** Expression of serum mtDNA genes (Cox1 and 12 S) in male wild type (WT) or MAVS KO mice exposed to no stress (NS) or restraint stress (RS). Cox1; One-way ANOVA, *p < 0.05 (vs WT-NS) and #*p* < 0.05 (vs WT-RS), *n* = 4 per group. 12 S; One-way ANOVA, *p < 0.05 (vs WT-NS) and #*p* < 0.05 (vs WT-RS), *n* = 4 per group. **C**, **D** MAVS deletion attenuated chronic stress-induced deficits in social behavior. **C** Time in chamber in the three-chamber social interaction test. Two-way ANOVA, chamber (F (2, 78) = 56.02, *P* < 0.0001); interaction (chamber X genotype) (F (4, 78) = 2.672, *P* = 0.0381). *p < 0.05 (mouse chamber vs empty chamber); *n* = 9–10 per group. **D** Reciprocal social interaction test; One-way ANOVA, *p < 0.05 vs WT-NS; *n* = 9–10 per group.

MAVS plays a key role in maintaining mitochondrial homeostasis via autophagy [55]. Activation of MAVS has been shown to recruit downstream signaling molecules and kinases, leading to the induction of type 1 interferon (IFN-I) proteins and other inflammatory cytokines [58–61, 69, 70]. Our earlier study has found significant increases in serum IFNβ levels in mice following RS, and systemic blockade of IFN-I signaling attenuated RS-induced social behavior deficits [28]. However, the role of MAVS in stress-induced changes in social behavior is not known. Here, we used MAVS KO mice to investigate MAVS function in RS-induced increases in cf-mtDNA levels and social behavior deficits. In male mice, MAVS deletion significantly attenuated RS-induced increases in serum Cox1 and 12 S levels (Fig. 3A, B). Furthermore, WT showed no preference for either chamber following RS, whereas MAVS KO mice exposed to RS showed a preference for the chamber housing stranger mouse than the empty cage chamber (Fig. 3C). Similarly, WT stressed mice showed decreased interaction with a stranger mouse compared to MAVS KO mice under RS exposure (Fig. 3D). Also, we found a significant attenuation in RS-induced social behavior deficits in female MAVS KO mice (Fig. S1). Overall, these results suggest that MAVS plays a key role in chronic stress-induced increases in cf-mtDNA levels and social behavior deficits.

### Chronic stress-stimulated mtDNA induces TLR9-dependent social behavior deficits

**Fig. 4:**
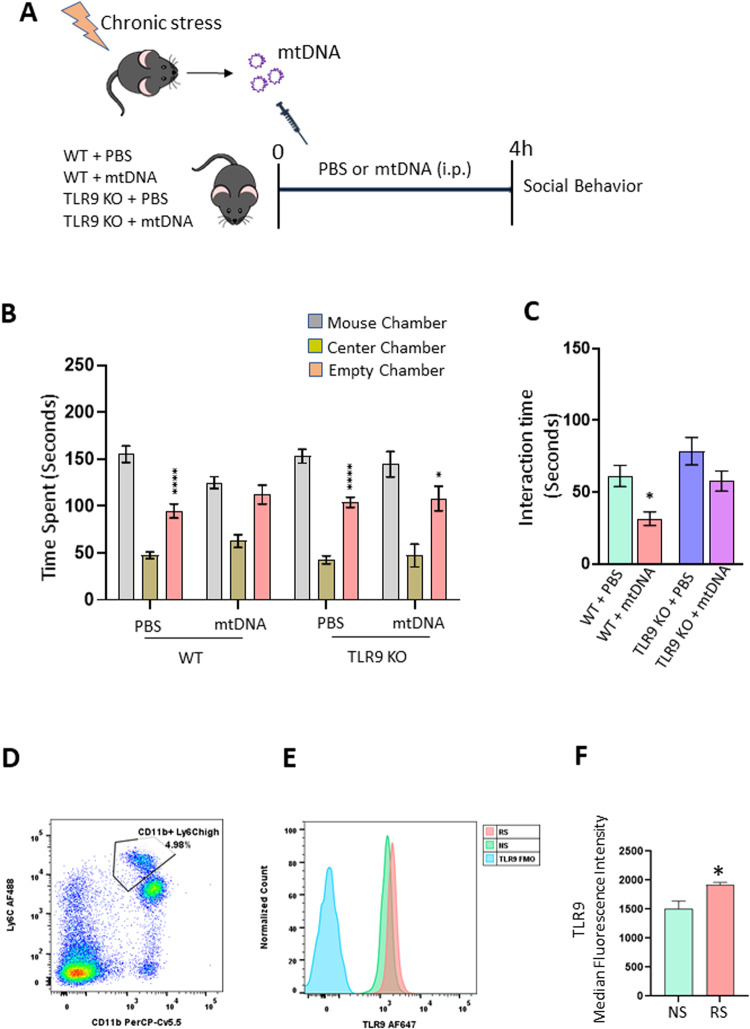
Chronic stress-stimulated mtDNA induces TLR9-dependent social behavior deficits. **A**–**C** TLR9 deletion attenuated mtDNA-induced deficits in social behavior. **A** Experimental plan. **B** Time in chamber in the three-chamber social interaction test. Two-way ANOVA, chamber (F (2, 99) = 132.8, *p* < 0.0001); interaction (chamber X treatment) (F (6, 99) = 2.609, *p* = 0.0217). ****p < 0.0001 and *p < 0.05 (mouse chamber vs empty chamber); *n* = 7–10 per group. **C** Reciprocal social interaction test; Two-way ANOVA, treatment (F (1, 32) = 10.55, *p* = 0.0027), genotype (F (1, 32) = 7.809, *p* = 0.0087). *p < 0.05 vs WT-PBS; *n* = 6–10 per group. **D**, **E** Flow cytometry analysis showing increased TLR9 expression in blood macrophages of mice exposed to restraint stress (RS). **D** Gating strategy for flow cytometry analysis for CD11b+ Ly6C^high^ cells. **E** TLR9 fluorescence intensity on gated CD11b+ Ly6C^high^ cells in the blood of no stress (NS) and restraint stress (RS) mice. **F** Quantified TLR9 median fluorescence intensity represented in bar graph. Student’s *t* test; *p < 0.05 vs NS; *n* = 4 per group.

To determine if mtDNA is sufficient to cause social behavior deficits under chronic stress conditions, we administered chronic stress-stimulated mtDNA into control mice and social behavior was examined. For this, we have purified mtDNA from RS mice and injected it into WT control mice (Fig. 4A). We found that stress-exposed mtDNA was able to induce social behavior deficits in the three-chamber sociability test (Fig. 4B) and social interaction test (Fig. 4C) in control mice. mtDNA are recognized by TLR9 resulting in the activation of proinflammatory signaling pathways [38]. To determine the role of TLR9 in mediating mtDNA-induced social behavior deficits, mtDNA isolated from RS-exposed mice were administered to TLR9 KO mice (Fig. 4A). We found that the absence of TLR9 attenuated mtDNA-induced social behavior deficits (Fig. 4B, C). Using flow cytometry analysis, we found that TLR9 expression is increased in macrophages following RS (Fig. 4D–F).

## Discussion

Using restraint stress model, we found that chronic stress increases cf-mtDNA levels and inhibition of MAVS attenuates chronic stress-induced social behavior deficits in mice. Furthermore, we showed that TLR9 plays a critical role in mediating mtDNA-induced social behavior deficits.

Social behavioral impairments are a characteristic of a number of neurodevelopmental as well as neuropsychiatric disorders, including autism spectrum disorder (ASD), schizophrenia (SCZ), and major depressive disorder (MDD). Moreover, disturbances of mitochondrial function are known to cause social behavior deficits [71, 72]. Increased mitochondrial activity and the associated reduced GABAergic transmission have recently been shown to result in social behavioral deficits [73]. The high energy consumption by the brain requires mitochondria to mobilize the metabolites via the tricarboxylic acid cycle and ATP through oxidative phosphorylation [71, 72, 74]. Mitochondria play an important role in brain plasticity including neurogenesis and neurotransmission [74–79]. Therefore, any disturbance in mitochondrial homeostasis can influence the neuronal activity and brain function including social behavior.

Increased levels of plasma cf-mtDNA have been reported in suicide attempters [45] and major depressive disorder (MDD) subjects [41]. cf-mtDNA is a part of mitochondrial damage-associated molecular patterns (DAMPs) which are capable of eliciting inflammatory responses. A number of mechanisms of cf-DNA release from cells have been proposed which are categorized into two: cell death and active secretion. On the other hand, cf-mtDNA clearance is facilitated by either cf-mtDNA degradation by circulating DNases [80] or cf-mtDNA uptake by target cells [81]. mtDNA can elicit various proinflammatory signaling pathways by TLR9, cGAS-STING or inflammasome pathway depending on their localization. Cytoslic mtDNA is recognized by cGAS resulting in the activation of endoplasmic reticulum (ER)-localized STING and an interferon response. Similarly, cytosolic mtDNA stimulates inflammasome activity leading to increased levels of proinflammatory IL-1 and IL-8. On the other hand, mtDNA released into the blood is recognized by TLR9. We found that systemic reduction of cf-mtDNA using DNase I significantly blocked RS-induced neuroinflammation and social behavior deficits. Although we found an increase in cf-mtDNA following chronic stress exposure, the cellular source of the mtDNA is not known. Also, it is important to note that our findings on the effects of DNase I on social behavior were derived from experiments using one dose of DNase I. Future studies should examine the effects of additional doses of DNase I and other approaches to deplete mtDNA on social behavior.

In the present study, we have used the RS paradigm to investigate the effects of chronic stress on cf-mtDNA-mediated changes in inflammatory markers and social behavior. A number of previous studies have shown the effects of RS on anxiety and depression-like behaviors [82–85], brain connectivity [86, 87], hippocampal volume [88, 89], social behavior [28, 90] and cognitive functions [91–95] similar to those observed in depressed subjects [96]. In addition, RS exposure in rodents resulted in persistent low-grade inflammation, as shown by increases in peripheral levels of proinflammatory markers [28, 97, 98]. Although other chronic stress models such as chronic unpredictable stress and social defeat stress are known to induce neuroinflammation and behavioral abnormalities their effects on cf-mtDNA are not known.

Mitophagy is a key quality control mechanism that selectively eliminates dysfunctional mitochondria through autophagy [99]. Impairments in clearing defective mitochondria via mitophagy leads to the accumulation of mtDNA and subsequent proinflammatory response [100]. A number of studies have suggested a potential role of peripheral mtDNA in linking mitochondrial dysfunction to systemic inflammation [101]. Although the exact mechanism of mtDNA release into the circulation under chronic stress conditions is currently unknown, we believe that glucocorticoid signaling plays a causative role in stress-induced changes in mitochondrial homeostasis. In this regard, an in vitro study in primary human fibroblasts has found increase in mtDNA extrusion following dexamethasone treatment [102]. The impairments in the mitophagy process can lead to oxidative stress and reduced membrane potential [103]. The damaged mitochondria from defective mitophagy could result in the extrusion of components such as mtDNA into the cytosol and to the circulation leading to sustained inflammation. The effects of RAPA, a mammalian target of rapamycin (mTOR) inhibitor on social behavior has been tested in a number of studies using animal models of ASD. RAPA treatment has been shown to recover social deficits in Tsc mutant [104] and Cntnap2 −/− [105] mice. In addition, RAPA improved valproic acid-induced social deficits in mice [106]. Our data show a protective effect of RAPA against chronic stress-induced social behavior deficits. A number of studies have examined the effects of chronic stress on mTOR signaling. Chronic restraint stress (CRS) in rats for 6 h daily for 21 days has been shown to increase mTOR levels in the hippocampus [107]. However, another study in mice exposed to CRS for 4 h daily for 21 days found a decrease in the levels of phospho-mTOR/mTOR in the hippocampus [108]. Moreover, activation of the mTOR signaling pathway in the PFC plays an important role in the antidepressant action of ketamine [109]. On the other hand, inhibition of mTOR attenuated cognitive deficits and reduced amyloid-beta levels in a mouse model of Alzheimer’s disease [110]. These studies suggest that a balance between mTOR activation and inhibition is critical for neuroplasticity, and the timing and duration of stress play important role in the outcome.

MAVS is located on the outer membrane of mitochondria (OMM) and is known to promote proinflammatory signaling pathways following viral infections [60]. Specifically, MAVS activation results in mitochondrial dysfunction and subsequent release of ROS and mtDNA into the cytosol [111]. A number of mechanisms such as protein–protein interactions, mitochondrial dynamics and post-translational modifications have been implicated in the regulation of the expression and/or signaling of MAVS [112]. One such mitochondrial protein which has been identified as a negative regulator of MAVS is the nucleotide-binding domain and leucine-rich repeat containing family member, NLRX1 [113]. NLRX1 is present at the OMM and inhibits MAVS interactions with its signaling partners [114]. For example, NLRX1 negatively regulates infection-induced IFN-I signaling and IL-6 production in primary MEFs by inhibiting the interaction between MAVS and RIG-I [115]. It should be noted that molecules such as mitofusin 2 [116] and translocase of outer membrane 70 (Tom70) [117] have also been identified as MAVS interaction proteins. Although our study found an inhibition of stress-induced increases in cf-mtDNA levels and social behavior deficits in MAVS KO mice, additional studies are needed to elucidate the role of MAVS binding partners in mediating stress-induced behavioral changes.

TLRs play important role in regulating innate immunity and inflammation. Among the different TLRs, TLR9 is the only receptor for detecting DNA as well as oligodeoxynucleotides containing the CpG motifs and therefore, the cell-free DNA released from mitochondria which is rich in unmethylated CpGs can trigger inflammation via TLR9 [38]. mtDNA binds to TLR9 through a sequence specific binding to the N-terminal of the C-shaped leucine-rich repeat region of TLR9 [118]. Binding of DNA molecules to each monomer results in the dimerization of TLR9 [119] and subsequent interaction with myeloid differentiation primary response 88 (MYD88) adapter protein [47]. It is known that mitogen activated protein kinase (MAPK) and nuclear factor kappa-light-chain-enhancer of activated B cells (NF-kB) pathways participate in the TLR9-mediated transcription of proinflammatory cytokines [47]. Although most of the previous studies have investigated the role of TLR9 expressed in immune cells, it is also expressed in non-immune cells including neurons [120]. An earlier study has shown that TLR9 deficiency blocks chronic stress-induced changes of serum proinflammatory cytokines in mice [121]. Furthermore, a deficiency in TLR9 attenuated stress-enhanced corticosterone levels suggesting that TLR9 is necessary for the upregulation of the corticosterone in response to chronic stress [122].

**Fig. 5:**
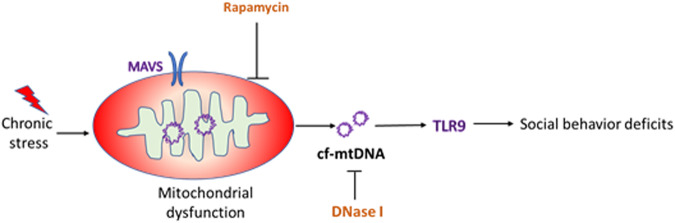
cf-mtDNA-TLR9 signaling mediates chronic stress-induced social behavior deficits. Mitochondrial antiviral-signaling protein (MAVS) plays a key role in maintaining mitochondrial homeostasis via autophagy. Rapamycin treatment and MAVS deletion attenuate chronic stress-induced increases in serum cf-mtDNA and social behavior deficits. In addition, Deoxyribonuclease I (DNase I) treatment attenuates chronic stress-induced social behavior deficits. mtDNA are recognized by Toll-like receptor 9 (TLR9) in macrophages resulting in the activation of proinflammatory signaling pathways and social behavior deficits.

In summary, we uncovered a novel mechanism of chronic stress-induced social behavior deficits and our findings showed that cf-mtDNA-TLR9 signaling is critical in mediating stress-induced social behavior deficits (Fig. 5). A limitation of the present study is that we used global KO mice and therefore, the cell type-specific role of TLR9 or MAVS in mediating stress-induced behavior changes is not known. We are currently investigating the role of neuronal vs immune cell-specific function of TLR9 in neuroplasticity and behavior. Molecules such as MAVS and mTOR are the key mediators of stress-induced mitochondrial abnormalities and therefore, compounds that modulate either these pathways or TLR9 may represent potential therapeutic approaches to treat social behavior deficits seen in many neuropsychiatric conditions.

### Supplementary information

Supplementary information

Table S1

Figure S1
