# Supplementary material for: Activation of cell-free mtDNA-TLR9 signaling mediates chronic stress-induced social behavior deficits
Source: Mol Psychiatry. 2023 Aug 1;28(9):3806–15. doi: 10.1038/s41380-023-02189-7 (PMC10730412; doi:10.1038/s41380-023-02189-7)
Supplement: Supplementary file 1 — Supplementary information [file 41380_2023_2189_MOESM1_ESM.docx]

**Supplementary Information:**

**Supplementary table 1.** Primer sequences

**Supplementary Figure S1.** MAVS deletion attenuated chronic stress-induced social behavior deficits in female mice. (**A**) Time in chamber in the three-chamber social interaction test. Two-way ANOVA, chamber (F (2, 72) = 32.04, P<0.0001). *p<0.05 (mouse chamber vs empty chamber); n=9 per group. (**B**) Reciprocal social interaction test; One-way ANOVA, *p<0.05 vs WT-NS; n=9 per group.
