# Supplementary material for: Activation of cell-free mtDNA-TLR9 signaling mediates chronic stress-induced social behavior deficits
Source: Mol Psychiatry. 2023 Aug 1;28(9):3806–15. doi: 10.1038/s41380-023-02189-7 (PMC10730412; doi:10.1038/s41380-023-02189-7)
Supplement: Supplementary file 2 — Table S1 [file 41380_2023_2189_MOESM2_ESM.docx]

| **Gene** | **Forward Primer (5’-3’)** | **Reverse Primer (5’-3’)** |
| --- | --- | --- |
| *12S* | ACCGCGGTCATACGATTAAC | CCCAGTTTGGGTCTTAGCTG |
| *Cox1* | GCCCCAGATATAGCATTCCC | GTTCATCCTGTTCCTGCTCC |
| *iNOS* | CCCTTCAATGGTTGGTACATG G | ACATTGATCTCCGTGACAGCC |
| *TNFα* | CGTCAGCCGATTTGC TATCT | CGGACTCCGCAAAGTCTAAG |
| *B2M* | CCCCACTGAGACTGATACATACG | CGATCCCAGTAGACGGTCTTG |

**Table 1. Mouse primer sequence**
