# Supplementary figures and images for: Activation of cell-free mtDNA-TLR9 signaling mediates chronic stress-induced social behavior deficits
Source: Mol Psychiatry. 2023 Aug 1;28(9):3806–15. doi: 10.1038/s41380-023-02189-7 (PMC10730412; doi:10.1038/s41380-023-02189-7)

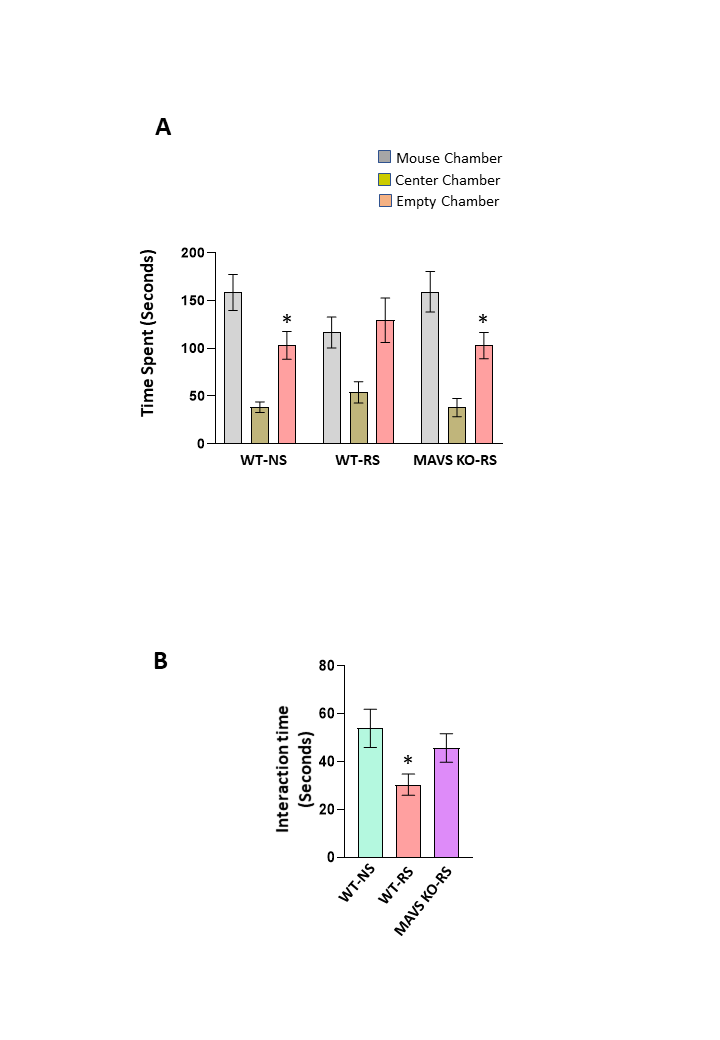

Supplement: Supplementary file 3 — Figure S1 [file 41380_2023_2189_MOESM3_ESM.tif]
